# Within-person reciprocal associations among physical activity, loneliness, and social anxiety in adolescence

**DOI:** 10.3389/fpsyg.2026.1805256

**Published:** 2026-07-02

**Authors:** Chang Hu, Wen Liu, Xi Wang, Xin Li, Kun Xu

**Affiliations:** 1 Professional Tennis College, Wuhan City Polytechnic, Wuhan, China; 2 Department of General Education, Wuhan College, Wuhan, China; 3 Department of Physical Education, Guangzhou College of Technology and Business, Guangzhou, China; 4 Department of Sociology, University of Manchester, Manchester, United Kingdom

**Keywords:** adolescents, Chinese, loneliness, physical activity, reciprocal association, RI-CLPM, social anxiety

## Abstract

**Introduction:**

Social anxiety (SA) is a common and developmentally significant concern during adolescence. Although prior research suggests that physical activity (PA), loneliness, and SA are correlated, less is known about how these associations develop over time. This study examined the within-person longitudinal associations among these variables in a sample of Chinese adolescents.

**Methods:**

Data were collected across four waves from 1,440 students (52.3% male; Mage = 13.39 years, *SD* = 0.62). A Random Intercept Cross-Lagged Panel Model was used to analyze the data.

**Results:**

At the within-person level, higher SA predicted subsequent increases in loneliness, while loneliness in turn predicted higher SA, suggesting a reciprocal and reinforcing process. Higher PA was associated with later decreases in both SA and loneliness, whereas SA and loneliness predicted lower subsequent PA.

**Conclusion:**

These findings highlight the dynamic links among SA, loneliness, and PA, underscoring the importance of addressing social–emotional difficulties while promoting PA to reduce adolescent loneliness.

## 1 Introduction

Social anxiety (SA) is a persistent emotional condition marked by intense fear and discomfort in actual or anticipated social situations (McNeil and Randall, 2014). Adolescents with high SA tend to engage in negative self-evaluation, excessive fear of rejection, and heightened sensitivity to others’ judgments. These tendencies are associated with avoidance behaviors and impaired daily functioning (Camacho et al., 2022; Dryman et al., 2016). Prolonged SA is linked with reduced quality of life and increased psychological distress (Ratnani, 2017; Stein and Kean, 2000). Recent research has increasingly focused on the associations among physical activity (PA), loneliness, and SA among adolescents. Although prior studies demonstrate associations among these variables, longitudinal within-person dynamics remain unclear. It is still unknown how these variables relate to one another across time. It is also uncertain whether these associations reflect stable between-person differences or true within-person processes. Understanding these distinctions is important for clarifying developmental patterns during adolescence. Based on these gaps, the following sections review existing evidence concerning the within-person bidirectional associations among PA, SA, and loneliness.

### 1.1 The association between PA and SA

According to Self-Determination Theory (SDT; Ng et al., 2012), PA is closely associated with the fulfillment of basic psychological needs for autonomy, competence, and relatedness. Adolescents taking part in PA, particularly in structured or group contexts, often report higher perceptions of competence through skill development, greater autonomy by engaging in activities aligned with personal interests, and stronger relatedness through connections with peers or teammates (Fraguela-Vale et al., 2020; Holt et al., 2019). When these needs are met, adolescents are more likely to experience psychological well-being, which is typically accompanied by lower levels of SA symptoms such as fear of negative evaluation and avoidance of social situations (Wu et al., 2025a). At the same time, SDT provides a framework for understanding how SA may be associated with reduced PA participation. Socially anxious adolescents often withdraw from evaluative or group contexts because of fear of scrutiny, which can limit opportunities to experience competence and relatedness in PA settings (Üstündağ et al., 2025). This pattern of avoidance may be associated with greater frustration of psychological needs, thereby maintaining both lower PA participation and higher SA (Wu et al., 2025a). In this way, SDT highlights a dynamic interplay in which PA and SA are connected through need satisfaction and need frustration.

Accumulating empirical evidence has consistently documented inverse associations between PA and SA. Cross-sectional findings suggest that individuals who participate more frequently in organized sports or regular exercise generally report lower levels of SA. For instance, recent studies have shown that higher levels of PA are associated with reduced SA among Chinese adolescents (Wu et al., 2025a) and college populations (Deng and Wang, 2024; Jiang et al., 2025; Li et al., 2024a,b; Shang et al., 2023). Moreover, a systematic review and meta-analysis concluded that PA demonstrates small yet promising associations with reductions in SA across multiple study designs, highlighting its potential as an adjunctive treatment for SA, although additional high-quality randomized controlled trials are still needed (Zika and Becker, 2021). Extending this evidence, longitudinal research indicates that the relationship between PA and SA may be dynamic and reciprocal. A recent multi-wave study conducted with Chinese adolescents found that higher levels of physical exercise were associated with reduced adolescent SA (Wu et al., 2025b). However, less is known about whether PA and SA predict each other at the within-person level over time.

### 1.2 The association between loneliness and SA

Loneliness is generally conceptualized as the aversive experience that arises when there is a perceived discrepancy between the desired and the actual quantity or quality of social relationships (Chen et al., 2025; Salari et al., 2025). Recent evidence indicates that loneliness is a prevalent issue among young people worldwide. A large-scale systematic review across 113 countries estimated that approximately 9.2–14.4% of adolescents experience frequent loneliness (Surkalim et al., 2022). Accumulating research further shows that loneliness does not occur in isolation; rather, it interacts closely with other socio-emotional difficulties, such as social anxiety. Loneliness and social anxiety may reinforce each other through a reciprocal cycle. As an evolutionarily shaped self-protective signal (Cacioppo and Cacioppo, 2018), loneliness heightens vigilance to potential social threats and fosters negatively biased interpretations of others’ intentions. This threat-oriented processing increases anticipatory anxiety in social situations, prompting withdrawal and other self-protective interpersonal behaviors (Cacioppo and Hawkley, 2009). Reduced social engagement then intensifies feelings of loneliness, which further amplifies threat sensitivity and social anxiety (Cacioppo et al., 2010). Over time, this mutually reinforcing loop may maintain and worsen both conditions.

Empirical evidence has consistently demonstrated a strong association between SA and loneliness. Cross-sectional findings repeatedly show this association across both adolescent and young adult samples (Chen and Hu, 2022; Eres et al., 2023; Hoffman et al., 2021). Moreover, individuals diagnosed with SA disorder consistently report greater loneliness than those without the disorder (Eres et al., 2020; Oren-Yagoda et al., 2022). A comprehensive meta-analysis confirmed these findings, demonstrating a strong positive cross-sectional association between SA and loneliness across childhood and adolescence, and further revealing significant reciprocal longitudinal associations between the two constructs (Maes et al., 2019). Longitudinal studies provide additional evidence. For example, a cross-lagged panel model found that SA symptoms significantly predicted higher loneliness 5 years later, although the reverse path was not significant after adjusting for covariates (Reinwarth et al., 2024). In Chinese left-behind children, loneliness and SA mutually predicted each other across time (Wu et al., 2024). Similarly, a growth modeling study showed that increases in loneliness were associated with parallel increases in SA during adolescence (Danneel et al., 2020). These findings highlight that SA and loneliness are closely associated over time.

### 1.3 The association between physical activity and loneliness

Based on SDT (Ng et al., 2012), participation in PA helps satisfy the basic psychological needs of autonomy, competence, and relatedness: adolescents gain a sense of control through self-directed engagement, develop competence via skill improvement, and fulfill relatedness through peer interaction and teamwork (Li et al., 2024a,b; Teixeira et al., 2012). These processes contribute to reduced loneliness and enhanced well-being (Su et al., 2024). Conversely, loneliness reflects unmet relatedness needs, which can undermine intrinsic motivation to be active (Broen et al., 2023). Adolescents who feel socially isolated may be less willing to join group-based activities, perceive themselves as less competent, and lack the social support required to sustain participation (Hawkley and Cacioppo, 2010). Such barriers lower PA levels and, in turn, restrict opportunities to meet psychological needs, thereby intensifying loneliness. Thus, PA and loneliness are not merely linked in one direction but interact in a reinforcing cycle, particularly salient during adolescence when peer connections are central to psychosocial development.

Accumulating empirical evidence consistently demonstrates a strong association between PA and loneliness. Cross-sectional studies conducted across diverse cultural contexts, including European (Lippke et al., 2021; Tubić et al., 2023), East Asian (Kwon and Jang, 2024), and Chinese samples (Zhao et al., 2024), repeatedly show that adolescents who participate more frequently in PA, particularly in organized or team-based activities, report lower levels of loneliness. Systematic reviews similarly conclude that PA is reliably associated with lower loneliness, particularly when activities emphasize social interaction, such as team or organized sports (Ahn et al., 2024; Pels and Kleinert, 2016).

Longitudinal evidence further suggests that the PA-loneliness relationship may be bidirectional. In a Norwegian cohort study, repeated measures before and after the pandemic showed that decreases in PA were accompanied by increases in loneliness, while more active adolescents were consistently less lonely (Rangul et al., 2024). Trajectory analyses also indicate that adolescents who maintained sport participation over time had a significantly lower risk of subsequent loneliness (Owen et al., 2024). In a large multi-wave panel of middle-aged and older American adults, higher PA at one time point predicted lower loneliness at the next, while higher loneliness predicted lower subsequent PA, thereby demonstrating a bidirectional association (Surkalim et al., 2024). These longitudinal studies suggest that PA not only protects against loneliness, but may also be inhibited by loneliness.

### 1.4 The present study

Despite the growing literature, important limitations remain. First, much of the existing evidence is based on cross-sectional designs, which restricts causal inference. Second, although longitudinal studies have begun to examine these associations, most rely on conventional cross-lagged models that do not adequately distinguish stable between-person differences from within-person fluctuations, making it unclear whether observed effects reflect genuine intraindividual change. Third, few studies have focused specifically on Chinese adolescents. Finally, few studies have examined PA, SA, and loneliness simultaneously. To address these gaps, the present study applies a Random Intercept Cross-Lagged Panel Model (RI-CLPM) with four waves of data from Chinese adolescents. This approach enables the disentangling of stable between-person differences from dynamic within-person processes, providing a more rigorous test of the reciprocal pathways linking PA, SA, and loneliness. Based on theoretical and empirical foundations, we hypothesized that, at the within-person level, (H1) PA and SA would be bidirectionally associated over time. (H2) SA and loneliness would be bidirectionally associated over time. (H3) PA and loneliness would be bidirectionally associated over time.

## 2 Methods

### 2.1 Participants and procedure

This longitudinal research was carried out over the course of 1 year (September 2023–March 2025) in six middle schools located in Hubei, China. Data were collected at four intervals, each 6 months apart: the first wave (T1) in September 2023, the second wave (T2) in March 2024, the third wave (T3) in September 2024, and the fourth wave (T4) in March 2025. Participants were recruited using convenience sampling. The six schools were not randomly selected; rather, they were selected based on accessibility, administrative permission, and willingness to participate in the four-wave longitudinal survey. Within each participating school, data were collected from intact classes during regular school hours, rather than through open voluntary recruitment. Trained research staff visited classrooms during regular school hours to administer self-report questionnaires.

Before the surveys were distributed, both students and their parents or legal guardians received detailed information outlining the objectives of the study, the data collection procedures, and assurances of confidentiality. Parents/guardians gave written informed consent, while students were informed that returning the completed survey would be taken as an indication of assent. Participation was entirely voluntary, and students were assured that refusal or withdrawal would not influence their school records or academic evaluation.

To link responses across the four measurement waves, students recorded their school ID numbers. These identifiers were kept in a secure file and subsequently removed from the dataset used for analysis to protect anonymity. Prior to completion, participants were provided with an information sheet describing the study’s aims, procedures, and their rights as participants. At baseline (T1), 1711 adolescents participated. By the final wave (T4), 1,440 students (52.3% males; Mage = 13.39 years, SD = 0.62) remained, resulting in an overall retention rate of 84.2%. The study protocol was reviewed and approved by the Ethics Committee of the General Education Department, Wuhan College (Approval No. 20230906).

### 2.2 Measures

#### 2.2.1 Demographic variables

Demographic variables were collected, including age, sex, academic performance, self-reported household income level, and single-parent family status. Sex was assessed using male and female categories, and participants were allowed to leave this item unanswered.

#### 2.2.2 Social anxiety (SA)

The SA was assessed using the Social Anxiety subscale of the Self-Consciousness Scale (Fenigstein et al., 1975). This subscale comprises six items rated on a 5-point Likert scale (1 = not at all like me to 5 = very much like me). An example item is, “I find it easy to talk to strangers.” Scores are summed, with higher totals reflecting greater SA. Prior validation studies have confirmed the scale’s reliability and validity among Chinese university students (Shek, 1994). The Cronbach’s *α* was 0.88 at T1, 0.91 at T2, 0.94 at T3, and 0.95 at T4.

#### 2.2.3 Loneliness

Loneliness was measured using the Chinese six-item short form of the UCLA Loneliness Scale (Xiao and Du, 2023). The instrument captures experiences such as lacking companionship, having no one to rely on, feeling excluded, being unhappy due to withdrawal, experiencing isolation, and perceiving others as present but not truly connected. Each item is rated on a four-point Likert scale ranging from 1 (never) to 4 (often), with higher scores reflecting greater loneliness. The Cronbach’s α was 0.85 at T1, 0.88 at T2, 0.91 at T3, and 0.92 at T4.

#### 2.2.4 Physical activity (PA)

Physical activity (PA) was assessed with the Physical Activity Rating Scale–3 (Liang, 1994), a short self-report tool consisting of three items that evaluate exercise intensity, duration, and frequency. Each item is rated on a 5-point scale, and a composite score is derived using the formula: intensity × (duration – 1) × frequency, yielding values between 0 and 100. Higher scores indicate greater engagement in PA. Previous research has supported the PARS-3 as a reliable and valid measure in Chinese populations (Yang et al., 2021). The Cronbach’s α was 0.84 at T1, 0.87 at T2, 0.90 at T3, and 0.91 at T4.

### 2.3 Data analysis

Chi-square tests (for categorical variables) and independent samples t-tests (for continuous variables) were conducted to test potential attrition bias. Skewness and kurtosis were examined for PA, SA, and loneliness at each wave to evaluate the distributional characteristics of the main continuous study variables. Pearson correlations were derived to investigate bivariate associations among the main variables. These initial analyses were conducted using SPSS (Version 26.0). Pairwise deletion was utilized for the correlational analyses.

Longitudinal invariance analysis was conducted for PA, SA, and loneliness across the four time points. Model fit was evaluated based on widely accepted criteria: Comparative Fit Index (CFI) ≥ 0.90, and Root Mean Square Error of Approximation (RMSEA) and Standardized Root Mean Square Residual (SRMR) values ≤ 0.08 (Cheung and Rensvold, 2002; McDonald and Ho, 2002). Metric and scalar invariance were determined based on ΔCFI ≤ 0.010 and ΔRMSEA ≤ 0.015 between nested models (Kline, 2016). The findings confirmed that both metric and scalar invariance were met, supporting the temporal consistency of the factor structures.

RI-CLPM was employed to examine the within-person associations among PA, SA, and loneliness. Prior to estimation, all continuous measures were grand-mean centered so that intercepts could be interpreted as reflecting average levels across the study period. The modeling process began with an unconstrained specification in which all autoregressive and cross-lagged effects were freely estimated. Next, equality constraints were imposed step by step: first on autoregressive and cross-lagged paths across the four waves, followed by fixing the variances and covariances of the random intercepts at T2, T3, and T4. These constrained models were compared with the unconstrained model to identify a more parsimonious specification without substantially reducing model fit. After determining the most parsimonious and best-fitting model, covariates were introduced as predictors of the latent intercepts to control for stable between-person differences in the key constructs.

Longitudinal invariance analysis and RI-CLPM were analyzed using Mplus version 8.3. Missing data in these models were addressed using Full Information Maximum Likelihood (FIML). Statistical significance was evaluated using the conventional criterion of *p* < 0.05.

## 3 Results

### 3.1 Attrition analyses

As presented in Table 1, no significant differences emerged between participants who remained in the study and those who were lost to follow-up on demographic characteristics or key study variables.

**Table 1:** Attrition analyses.

Categorical variables	Follow-up		Lost to follow-up		*p*
	(*n* = 1,440)		(*n* = 271)		
	*n*	%	*n*	%	
Sex					0.713
Male	753	52.3	146	53.9	
Female	683	47.4	125	46.1	
Not reported	4	0.3	/	/	
Self-reported academic performance					0.083
Very poor	191	13.3	50	18.5	
Poor	284	19.7	60	22.1	
Average	379	26.3	61	22.5	
Good	329	22.8	62	22.9	
Very good	257	17.8	38	14.0	
Self-reported household income level					0.628
Very low	26	1.8	4	1.5	
Low	177	12.3	33	12.2	
Average	937	65.1	185	68.3	
High	278	19.3	43	15.9	
Excellent	22	1.5	6	2.2	
Single-parent family status					0.795
Yes	96	6.7	17	6.30	
No	1,168	81.1	217	80.1	
Not reported	176	12.2	37	13.7	

Continuous variables	Mean	SD	Mean	SD	*p*
Age	13.39	0.62	13.41	0.63	0.632
PA	43.16	35.69	42.23	34.18	0.684
SA	16.00	5.34	16.20	5.34	0.572
Loneliness	9.16	4.49	9.06	4.20	0.745

PA, physical activity; SA, social anxiety.

### 3.2 Distributional characteristics

As shown in Supplementary Table 1, skewness and kurtosis were examined for PA, SA, and loneliness at each measurement wave. The skewness values ranged from −0.19 to 1.49, and the kurtosis values ranged from −1.24 to 1.48. These distributions did not indicate severe deviations from normality for the main continuous variables.

### 3.3 Pearson correlations

As shown in Table 2, physical activity (PA) at T1-T4 was negatively associated with SA and loneliness across all four measurement waves, with correlation coefficients ranging from −0.48 to −0.22 (all *p*s < 0.001). In contrast, SA and loneliness were positively linked over time, with correlations between 0.32 and 0.58 (all *p*s < 0.001).

**Table 2:** Correlations.

Variable	1	2	3	4	5	6	7	8	9	10	11	12
PA at T1	1											
PA at T2	0.52^***^	1										
PA at T3	0.44^***^	0.50^***^	1									
PA at T4	0.37^***^	0.45^***^	0.54^***^	1								
SA at T1	−0.42^***^	−0.38^***^	−0.29^***^	−0.24^***^	1							
SA at T2	−0.33^***^	−0.48^***^	−0.36^***^	−0.30^***^	0.58^***^	1						
SA at T3	−0.26^***^	−0.37^***^	−0.46^***^	−0.35^***^	0.48^***^	0.62^***^	1					
SA at T4	−0.23^***^	−0.29^***^	−0.34^***^	−0.37^***^	0.45^***^	0.54^***^	0.62^***^	1				
Loneliness at T1	−0.40^***^	−0.32^***^	−0.25^***^	−0.27^***^	0.57^***^	0.41^***^	0.32^***^	0.29^***^	1			
Loneliness at T2	−0.26^***^	−0.38^***^	−0.27^***^	−0.33^***^	0.42^***^	0.54^***^	0.40^***^	0.37^***^	0.57^***^	1		
Loneliness at T3	−0.25^***^	−0.29^***^	−0.40^***^	−0.34^***^	0.36^***^	0.43^***^	0.55^***^	0.52^***^	0.48^***^	0.55^***^	1	
Loneliness at T4	−0.22^***^	−0.27^***^	−0.31^***^	−0.36^***^	0.35^***^	0.47^***^	0.54^***^	0.56^***^	0.43^***^	0.50^***^	0.59^***^	1

***p < 0.001, PA, physical activity; SA, social anxiety.

### 3.4 Longitudinal invariance test

Table 3 shows the longitudinal measurement invariance results for PA, SA, and loneliness. The configural models showed acceptable fit (CFI > 0.90; RMSEA/SRMR < 0.08), indicating stable factor structures across the four waves. Both metric and scalar invariance were supported, as changes in fit indices (ΔCFI ≤ 0.01; ΔRMSEA ≤ 0.015) remained within recommended thresholds. These results indicate that the measures were consistent over time, allowing meaningful comparisons of scores across waves.

**Table 3:** Longitudinal invariance test.

Variable	Model	CFI	RMSEA	SRMR	ΔCFI	ΔRMSEA	ΔSRMR
PA	Configural Invariance	0.949	0.060	0.026	–	–	–
	Metric Invariance	0.949	0.053	0.029	0.000	0.007	0.003
	Scalar Invariance	0.945	0.050	0.035	0.004	0.003	0.006
SA	Configural Invariance	0.958	0.049	0.026	–	–	–
	Metric Invariance	0.957	0.048	0.029	0.001	0.001	0.003
	Scalar Invariance	0.954	0.049	0.029	0.003	0.001	0.000
Loneliness	Configural Invariance	0.959	0.053	0.034	–	–	–
	Metric Invariance	0.958	0.058	0.036	0.001	0.005	0.002
	Scalar Invariance	0.957	0.054	0.036	0.001	0.004	0.000

PA, physical activity; SA, social anxiety.

### 3.5 RI-CLPM results

**Figure 1:**
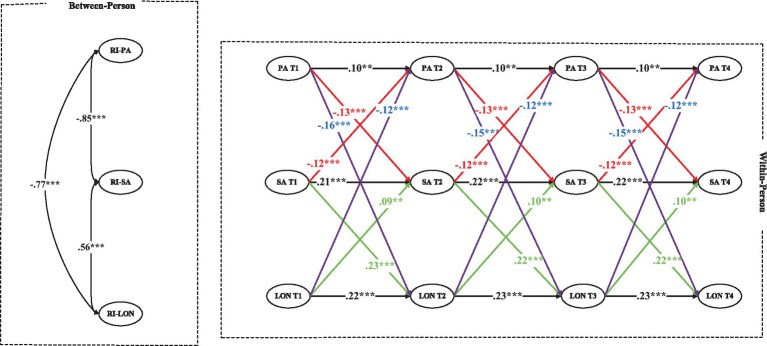
The reciprocal associations between PA, social anxiety, and loneliness. **p < 0.01, ***p < 0.001, PA, physical activity; SA, social anxiety; LON, loneliness.

RI-CLPM showed an acceptable model fit to the data: χ^2^(85) = 496.79, CFI = 0.931, TLI = 0.904, RMSEA = 0.067, and SRMR = 0.054. Detailed parameter estimates are demonstrated in Figure 1. With respect to within-person stability, all autoregressive coefficients were statistically significant, indicating that individual differences in PA, SA, and loneliness exhibited temporal continuity across adjacent waves.

Regarding cross-lagged effects, PA negatively predicted subsequent SA (T1–T2: *β* = −0.13, *p* < 0.001; T2–T3: *β* = −0.13, *p* < 0.001; T3–T4: *β* = −0.13, *p* < 0.001) and loneliness (T1–T2: *β* = −0.16, *p* < 0.001; T2–T3: *β* = −0.15, *p* < 0.001; T3–T4: *β* = −0.15, *p* < 0.001). Conversely, both SA (T1–T2: *β* = −0.12, *p* < 0.001; T2–T3: *β* = −0.12, *p* < 0.001; T3–T4: *β* = −0.12, *p* < 0.001) and loneliness significantly predicted PA (T1–T2: *β* = −0.12, *p* < 0.001; T2–T3: *β* = −0.12, *p* < 0.001; T3–T4: *β* = −0.12, *p* < 0.001). Moreover, SA significantly predicted loneliness (T1–T2: *β* = 0.23, *p* < 0.001; T2–T3: *β* = 0.22, *p* < 0.001; T3–T4: *β* = 0.22, *p* < 0.001), while loneliness also predicted increases in SA (T1–T2: *β* = 0.09, *p* = 0.005; T2-T3: *β* = 0.10, *p* = 0.004; T3–T4: *β* = 0.10, *p* = 0.004).

Within-person residual correlations indicated that PA was negatively associated with both SA and loneliness at all four time points, with standardized coefficients ranging from −0.23 to −0.19 (all *p*s < 0.001). In contrast, SA and loneliness were positively correlated at the within-person level across waves, with coefficients ranging from 0.24 to 0.38 (all *p*s < 0.001).

At the between-person level, latent trait correlations further supported these associations. The latent factor of PA was strongly and negatively associated with the latent traits of SA (*r* = −0.85, *p* < 0.001) and loneliness (*r* = −0.77, *p* < 0.001). Additionally, SA and loneliness were strongly and positively associated at the between-person level (*r* = 0.56, *p* < 0.001).

## 4 Discussion

This study used a four-wave RI-CLPM to disentangle the within-person effects among PA, loneliness, and SA among Chinese adolescents. At the within-person level, higher PA was followed by lower levels of SA and loneliness, whereas higher SA or loneliness was followed by lower subsequent PA. SA and loneliness also predicted each other. The findings suggest that these three variables are dynamically linked over time. Our results provide evidence that social–emotional difficulties may not only be shaped by low PA but may also undermine adolescents’ subsequent engagement in PA. This reciprocal pattern has implications for designing interventions that address both PA participation and social–emotional barriers.

### 4.1 The reciprocal constraint between PA and SA

The significant negative within-person effect of PA on later SA and the reverse effect of SA on subsequent PA support H1. The finding that short-term increases in PA predict later reductions in SA illustrates the role of PA as both a natural behavioral exposure and a context for psychological need fulfillment. Engaging in PA offers adolescents repeated opportunities to face low-stakes social interactions and mild evaluative situations (e.g., being observed by peers, receiving feedback from coaches), which can gradually reduce fear of scrutiny and challenge maladaptive avoidance patterns (Wu et al., 2025b). From a motivational perspective, PA also generates objective experiences of success, such as measurable improvements in stamina, skill acquisition, and mastery of tasks, which strengthen physical self-concept and more general feelings of competence (Zhang and Miao, 2024). These experiences fulfill the basic psychological need for competence, while autonomously chosen or intrinsically motivated PA simultaneously reinforces autonomy (Ntoumanis and Moller, 2025). When adolescents experience themselves as competent and self-directed in PA contexts, they may become less dependent on external approval and more resilient to negative evaluation in broader social situations (Wan et al., 2025). This can attenuate core features of SA such as fear of judgment and self-focused attention. In this way, PA may not only function as a graded, everyday form of social exposure, but also support self-beliefs and motivational orientations that are less compatible with persistent social fear.

The reverse within-person effect, whereby heightened SA predicts lower PA at the following wave, highlights that this association is embedded in a self-limiting cycle. For socially anxious adolescents, PA is not necessarily a neutral or inviting context; instead, it can be construed as a highly evaluative arena in which one’s body, skills, and performance are visible and potentially judged (Beenen et al., 2025; Plessner et al., 2023). Such contexts may be especially threatening for youths who already fear negative evaluation, making even routine or recreational activities feel like high-stakes tests of adequacy. Anticipated competence frustration and amplified self-presentational concerns can foster avoidance of PA as a defensive strategy to minimize exposure to perceived threat (Beenen et al., 2025). Although this withdrawal offers short-term relief from anxiety, it ultimately deprives adolescents of opportunities to satisfy competence, autonomy, and relatedness needs in PA settings (Back et al., 2022). Over time, this pattern creates a paradoxical loop: by avoiding PA to protect themselves from potential embarrassment or failure, socially anxious adolescents reduce access to precisely those experiences that could build self-efficacy, foster positive peer interactions, and gradually weaken SA. In line with recent studies showing that physical exercise is associated with lower adolescent SA and related psychosocial mechanisms, such as sports self-efficacy, expressive suppression, and psychological resilience (Wu et al., 2025a, 2025b), the present findings further suggest that the PA–SA association operates as a reciprocal within-person process after separating stable between-person differences. Thus, socially anxious adolescents may not only benefit from PA but may also become less likely to engage in PA when anxiety increases.

### 4.2 The vicious cycle of SA and loneliness

The reciprocal within-person association between SA and loneliness supports H2, indicating that elevated anxiety and heightened social disconnection mutually reinforce one another over time. The within-person path from higher SA to later loneliness was especially pronounced, underscoring the social cost of anxiety-driven avoidance. From an SDT perspective, SA shifts adolescents from autonomous engagement to fear-based, controlled motivation (Ryan and Deci, 2020, 2022), fostering anticipatory concerns about judgment and discouraging authentic participation in peer interactions (Chiu et al., 2021). This withdrawal reduces opportunities for reciprocal social exchanges and obstructs satisfaction of the relatedness need, which is particularly salient during adolescence (Pickering et al., 2020). The resulting erosion of meaningful social bonds may intensify perceived social isolation, producing the subsequent increases in loneliness observed in this study. This finding extends cross-sectional research by demonstrating temporal precedence: anxiety is not merely co-occurring with loneliness but tends to precede increases in loneliness at the intraindividual level.

Conversely, the reverse within-person pathway, where higher loneliness predicted increased SA at the next wave, reveals the competence- and threat-related implications of persistent social disconnection. Loneliness deprives adolescents of repeated social practice, limiting opportunities to strengthen communication skills, receive positive feedback, or build a sense of social efficacy (Lodder et al., 2016; Tella et al., 2023). This lack of reinforcement frustrates the need for competence and amplifies uncertainty in future social encounters (Chau et al., 2023). At the cognitive level, loneliness heightens hypervigilance to potential rejection and biases interpretations toward social threat (Qualter et al., 2013), which, in turn, elevates anticipatory anxiety and avoidance tendencies. Although this effect was smaller than the SA-to-loneliness pathway, its persistence demonstrates that loneliness is not merely a downstream outcome of anxiety but also a developmental risk factor that may exacerbate SA by constraining competence-building opportunities and reinforcing threat-sensitive processing (Maes et al., 2019). This pattern is consistent with prior longitudinal and meta-analytic evidence indicating close links between SA and loneliness during adolescence (Maes et al., 2019; Wu et al., 2024). The present findings extend this literature by showing that the association is not limited to between-person differences; rather, changes in SA and loneliness within the same adolescent also predict one another over time.

### 4.3 The reciprocal association between PA and loneliness

The reciprocal within-person association between PA and loneliness supports H3. The negative within-person cross-lagged path from PA to subsequent loneliness highlights the protective function of PA in adolescents’ social–emotional development. From the perspective of SDT, PA settings, particularly structured or organized activities that are common in Chinese schools, may help satisfy the need for relatedness (Ryan and Deci, 2020, 2022). Cooperative play, teamwork, and shared goals allow adolescents to experience belonging, mutual recognition, and social acceptance (White et al., 2021). These repeated social encounters help adolescents view themselves as valued members of a group, counteracting the perceived social disconnection central to loneliness (Eather et al., 2023; Tubić et al., 2023). The within-person nature of this effect suggests that increases in PA relative to an individual’s typical level are followed by measurable reductions in loneliness, indicating that PA may function as a short-term social–emotional resource rather than merely a stable lifestyle correlate (Belcher et al., 2021). By demonstrating temporal precedence, the findings extend cross-sectional evidence and provide stronger support for PA as a context that may facilitate social–emotional adjustment. Encouraging consistent PA participation may therefore serve as an accessible and non-stigmatizing approach to buffer adolescents against loneliness (Shvedko et al., 2018).

Recent longitudinal evidence suggests that sustained sport participation is associated with lower subsequent loneliness among young people, while panel evidence from adult samples also points to bidirectional links between PA and loneliness (Owen et al., 2024; Surkalim et al., 2024). The present findings extend this literature by showing that, among Chinese adolescents, loneliness is not only an outcome associated with lower PA but may also contribute to later reductions in PA at the within-person level. This bidirectional effect highlights a self-reinforcing cycle, suggesting that interventions should not only promote PA but also address the psychological barriers introduced by loneliness (Linardakis et al., 2025; Marciano et al., 2022). Creating socially safe, autonomy-supportive entry points for PA may help reduce avoidance tendencies and re-establish opportunities for positive social connection (Grenier et al., 2024; Chen et al., 2023).

### 4.4 Implications

Taken together, these findings extend previous work by showing that PA, SA, and loneliness form a dynamic within-person system. This pattern is particularly meaningful during adolescence, a developmental period marked by increasing academic pressure, sensitivity to peer evaluation, and evolving social expectations. Such demands may foster controlled forms of motivation and fragile social connections, making adolescents more vulnerable to anxiety and loneliness. The observed associations between lower PA and higher socio-emotional difficulties suggest that performance-focused or highly structured environments may hinder the fulfillment of basic psychological needs. In this context, PA can function as an important but often overlooked means of restoring autonomy, competence, and social connection. For educational practice, these results highlight the value of need-supportive interventions. Programs designed for anxious or lonely youth should: (1) validate emotional experiences to enhance autonomy; (2) provide meaningful choices and emphasize personal mastery to strengthen competence; and (3) incorporate non-evaluative, cooperative activities to foster relatedness. Future research should include direct assessments of psychological need satisfaction and frustration to clarify their role as mediating mechanisms within longitudinal models.

### 4.5 Limitations and future research

Despite its strengths, several limitations should be acknowledged. First, all variables were assessed through self-report questionnaires, which may introduce reporting bias. Future work should incorporate objective PA measures (e.g., accelerometry) and multi-informant reports. Second, although the four-wave design provides developmental insight, the six-month interval between waves may not fully capture short-term or non-linear dynamics. Intensive longitudinal methods, such as ecological momentary assessment or weekly diaries, could reveal finer-grained reciprocal processes. Third, although the RI-CLPM distinguishes within- from between-person effects, the design remains correlational and cannot establish causality. In addition, unmeasured time-varying factors, such as academic stress, peer relationship changes, or school-level activity opportunities, may have influenced the observed within-person associations. Experimental or quasi-experimental studies that manipulate PA opportunities or reduce social-evaluative threat would provide stronger causal evidence. Finally, the sample was drawn from six middle schools in one province of China, and the schools were selected through convenience sampling rather than random sampling. This sampling strategy may limit the representativeness of the sample and the generalizability of the findings. Cross-cultural research and studies using probability-based or more diverse samples are needed to test whether associations among PA, SA, and loneliness vary across educational environments or peer norms that differ in competitiveness or opportunities for organized activity.

## 5 Conclusion

This study examined the dynamic associations among PA, SA, and loneliness in adolescence using a four-wave RI-CLPM. Results showed clear within-person reciprocity across all constructs. Higher-than-usual PA predicted later decreases in both SA and loneliness. In contrast, increases in SA or loneliness predicted subsequent reductions in PA. SA and loneliness also predicted each other. These findings indicate that PA may help buffer short-term social–emotional difficulties during adolescence. They also suggest that rising SA or loneliness can undermine motivation to stay active. By isolating moment-to-moment fluctuations, the study demonstrates how daily experiences shape developmental trajectories. These findings underscore the importance of interventions that provide socially safe, autonomy-supportive PA settings. Programs that enhance adolescents’ confidence and reduce social-evaluative concerns may help disrupt these maladaptive cycles.

## Data Availability

The raw data supporting the conclusions of this article will be made available by the authors, without undue reservation.

## References

[ref1] Ahn J. Falk E. B. Kang Y. (2024). Relationships between physical activity and loneliness: a systematic review of intervention studies. Curr. Res. Behav. Sci. 6:100141. doi: 10.1016/j.crbeha.2023.100141

[ref2] Back J. Johnson U. Svedberg P. McCall A. Ivarsson A. (2022). Drop-out from team sport among adolescents: a systematic review and meta-analysis of prospective studies. Psychol. Sport Exerc. 61:102205. doi: 10.1016/j.psychsport.2022.102205

[ref3] Beenen K. T. Vosters J. A. Patel D. R. (2025). Sport-related performance anxiety in young athletes: a clinical practice review. Transl. Pediatr 14, 127–138. doi: 10.21037/tp-24-258, 39944878 PMC11811592

[ref4] Belcher B. R. Zink J. Azad A. Campbell C. E. Chakravartti S. P. Herting M. M. (2021). The roles of physical activity, exercise, and fitness in promoting resilience during adolescence: effects on mental well-being and brain development. Biol. Psychiatry 6, 225–237. doi: 10.1016/j.bpsc.2020.08.005, 33067166 PMC7878276

[ref5] Broen T. Choi Y. Zambrano Garza E. Pauly T. Gerstorf D. Hoppmann C. A. (2023). Time-varying associations between loneliness and physical activity: evidence from repeated daily life assessments in an adult lifespan sample. Front. Psychol. 13:1021863. doi: 10.3389/fpsyg.2022.1021863, 36778170 PMC9909092

[ref6] Cacioppo J. T. Cacioppo S. (2018). “Loneliness in the modern age: an evolutionary theory of loneliness (ETL),” in Advances in Experimental Social Psychology, ed. Olson J. M. (Cambridge, MA: Elsevier Academic Press), 127–197.

[ref7] Cacioppo J. T. Hawkley L. C. (2009). Perceived social isolation and cognition. Trends Cogn. Sci. 13, 447–454. doi: 10.1016/j.tics.2009.06.005, 19726219 PMC2752489

[ref8] Cacioppo J. T. Hawkley L. C. Thisted R. A. (2010). Perceived social isolation makes me sad: 5-year cross-lagged analyses of loneliness and depressive symptomatology in the Chicago health, aging, and social relations study. Psychol. Aging 25, 453–463. doi: 10.1037/a0017216, 20545429 PMC2922929

[ref9] Camacho A. Ortega-Ruiz R. Romera E. M. (2022). Adolescents’ social anxiety dynamics in a latent transition analysis and its psychosocial effects. Int. J. Clin. Health Psychol. 22:100311. doi: 10.1016/j.ijchp.2022.100311, 35662788 PMC9156938

[ref10] Chau A. K. C. So S. H. Barkus E. (2023). The role of loneliness and negative schemas in the moment-to-moment dynamics between social anxiety and paranoia. Sci. Rep. 13:20775. doi: 10.1038/s41598-023-47912-0, 38008774 PMC10679161

[ref11] Chen C. Hu L. (2022). Self-esteem mediated relations between loneliness and social anxiety in Chinese adolescents with left-behind experience. Front. Psychol. 13:1014794. doi: 10.3389/fpsyg.2022.1014794, 36425838 PMC9679498

[ref12] Chen C. Tahamata V. M. Chuang Y.-F. Huang Y.-S. Chuang Y.-H. Lin Y.-T. . (2025). Perceived loneliness mediates the relationship between mild cognitive impairment and executive function deficits. Humanit. Soc. Sci. Commun. 12:991. doi: 10.1057/s41599-025-05367-w

[ref13] Chen L. Zeng S. Su Y. (2023). The influence of social exclusion on adolescents’ social withdrawal behavior: the moderating role of connectedness to nature. J. Environ. Psychol. 87, 101951–101913. doi: 10.1016/j.jenvp.2022.101951, 38826717

[ref14] Cheung G. W. Rensvold R. B. (2002). Evaluating goodness-of-fit indexes for testing measurement invariance. Struct. Equ. Model. 9, 233–255. doi: 10.1207/S15328007SEM0902_5

[ref15] Chiu K. Clark D. M. Leigh E. (2021). Prospective associations between peer functioning and social anxiety in adolescents: a systematic review and meta-analysis. J. Affect. Disord. 279, 650–661. doi: 10.1016/j.jad.2020.10.055, 33190116 PMC7758784

[ref16] Danneel S. Geukens F. Maes M. Bastin M. Bijttebier P. Colpin H. . (2020). Loneliness, social anxiety symptoms, and depressive symptoms in adolescence: longitudinal distinctiveness and correlated change. J. Youth Adolesc. 49, 2246–2264. doi: 10.1007/s10964-020-01315-w, 32918664

[ref17] Deng Y. Wang X. (2024). The impact of physical activity on social anxiety among college students: the chain mediating effect of social support and psychological capital. Front. Psychol. 15:1406452. doi: 10.3389/fpsyg.2024.1406452, 38957885 PMC11217649

[ref18] Dryman M. T. Gardner S. Weeks J. W. Heimberg R. G. (2016). Social anxiety disorder and quality of life: how fears of negative and positive evaluation relate to specific domains of life satisfaction. J. Anxiety Disord. 38, 1–8. doi: 10.1016/j.janxdis.2015.12.003, 26709747

[ref19] Eather N. Wade L. Pankowiak A. Eime R. (2023). The impact of sports participation on mental health and social outcomes in adults: a systematic review and the ‘mental health through sport’ conceptual model. Syst. Rev. 12:102. doi: 10.1186/s13643-023-02264-8, 37344901 PMC10286465

[ref20] Eres R. Lim M. H. Bates G. (2023). Loneliness and social anxiety in young adults: the moderating and mediating roles of emotion dysregulation, depression and social isolation risk. Psychol. Psychother. 96, 793–810. doi: 10.1111/papt.12469, 37096794

[ref21] Eres R. Lim M. H. Lanham S. Jillard C. Bates G. (2020). Loneliness and emotion regulation: implications of having social anxiety disorder. Aust. J. Psychol. 73, 46–56. doi: 10.1111/ajpy.12296

[ref22] Fenigstein A. Scheier M. F. Buss A. H. (1975). Public and private self-consciousness: assessment and theory. J. Consult. Clin. Psychol. 43, 522–527. doi: 10.1037/h0076760

[ref23] Fraguela-Vale R. Varela-Garrote L. Carretero-García M. Peralbo-Rubio E. M. (2020). Basic psychological needs, physical self-concept, and physical activity among adolescents: autonomy in focus. Front. Psychol. 11:491. doi: 10.3389/fpsyg.2020.00491, 32265796 PMC7100532

[ref24] Grenier S. Gagné M. O’Neill T. (2024). Self-determination theory and its implications for team motivation. Appl. Psychol. 73, 1833–1865. doi: 10.1111/apps.12526

[ref25] Hawkley L. C. Cacioppo J. T. (2010). Loneliness matters: a theoretical and empirical review of consequences and mechanisms. Ann Behav Med 40, 218–227. doi: 10.1007/s12160-010-9210-8, 20652462 PMC3874845

[ref26] Hoffman Y. S. G. Grossman E. S. Bergman Y. S. Bodner E. (2021). The link between social anxiety and intimate loneliness is stronger for older adults than for younger adults. Aging Ment. Health 25, 1246–1253. doi: 10.1080/13607863.2020.1774741, 32524829

[ref27] Holt A.-D. Smedegaard S. Pawlowski C. S. Skovgaard T. Christiansen L. B. (2019). Pupils’ experiences of autonomy, competence and relatedness in ‘move for well-being in schools’: a physical activity intervention. Eur. Phys. Educ. Rev. 25, 640–658. doi: 10.1177/1356336X18758353

[ref28] Jiang Y. Zhang B. Zhao H. (2025). Analysing the effect of physical exercise on social anxiety in college students using a chained mediation model. Sci. Rep. 15:2475. doi: 10.1038/s41598-025-87140-2, 39833375 PMC11756402

[ref29] Kline R. B. (2016). Principles and Practice of Structural Equation Modeling, 4 (17–534). The Guilford Press. Available online at: https://psycnet.apa.org/record/2015-56948-000 (Accessed July 17, 2025).

[ref30] Kwon J. Jang J. (2024). The associations between the number of school sports teams that a student regularly participates in and factors such as perceived stress, loneliness, and sleep satisfaction among Korean adolescents who have attempted suicide. Children 11:77. doi: 10.3390/children11010077, 38255390 PMC10813959

[ref31] Li X. Liu Y. Rong F. Wang R. Li L. Wei R. . (2024a). Physical activity and social anxiety symptoms among Chinese college students: a serial mediation model of psychological resilience and sleep problems. BMC Psychol. 12:440. doi: 10.1186/s40359-024-01937-w, 39138553 PMC11323702

[ref32] Li X. Wang J. Yu H. Liu Y. Xu X. Lin J. . (2024b). How does physical activity improve adolescent resilience? Serial indirect effects via self-efficacy and basic psychological needs. PeerJ 12:e17059. doi: 10.7717/peerj.17059, 38436018 PMC10909365

[ref33] Liang D. (1994). The relationship between stress level and physical exercise for college students. Chin. Ment. Health J. 8, 5–6.

[ref34] Linardakis M. Vlachopoulos N. Mantadaki A. E. Mourellou E. Volkos P. Smyrnakis E. . (2025). Internet use and its association to physical activity, nutrition habits, sense of loneliness, and self-efficacy in Greek adolescents: a multi-center, school-based, cross-sectional study. Popul. Med. 7, 1–9. doi: 10.18332/popmed/210327

[ref35] Lippke S. Fischer M. A. Ratz T. (2021). Physical activity, loneliness, and meaning of friendship in young individuals – a mixed-methods investigation prior to and during the COVID-19 pandemic with three cross-sectional studies. Front. Psychol. 12:617267. doi: 10.3389/fpsyg.2021.617267, 33603702 PMC7884761

[ref36] Lodder G. M. A. Goossens L. Scholte R. H. J. Engels R. C. M. E. Verhagen M. (2016). Adolescent loneliness and social skills: agreement and discrepancies between self-, Meta-, and peer-evaluations. J. Youth Adolesc. 45, 2406–2416. doi: 10.1007/s10964-016-0461-y, 27071947 PMC5101254

[ref37] Maes M. Nelemans S. A. Danneel S. Fernández-Castilla B. Van den Noortgate W. Goossens L. . (2019). Loneliness and social anxiety across childhood and adolescence: multilevel meta-analyses of cross-sectional and longitudinal associations. Dev. Psychol. 55, 1548–1565. doi: 10.1037/dev0000719, 30896228

[ref38] Marciano L. Viswanath K. Morese R. Camerini A.-L. (2022). Screen time and adolescents’ mental health before and after the COVID-19 lockdown in Switzerland: a natural experiment. Front. Psych. 13:981881. doi: 10.3389/fpsyt.2022.981881, 36465307 PMC9709147

[ref39] McDonald R. P. Ho M.-H. R. (2002). Principles and practice in reporting structural equation analyses. Psychol. Methods 7, 64–82. doi: 10.1037/1082-989X.7.1.64, 11928891

[ref40] McNeil D. W. Randall C. L. (2014). “Chapter 1—conceptualizing and describing social anxiety and its disorders,” in Social Anxiety, eds. Hofmann S. G. DiBartolo P. M.. 3rd ed (San Diego, CA: Academic Press), 3–26.

[ref41] Ng J. Y. Y. Ntoumanis N. Thøgersen-Ntoumani C. Deci E. L. Ryan R. M. Duda J. L. . (2012). Self-determination theory applied to health contexts: a Meta-analysis. Perspect. Psychol. Sci. 7, 325–340. doi: 10.1177/1745691612447309, 26168470

[ref42] Ntoumanis N. Moller A. C. (2025). Self-determination theory informed research for promoting physical activity: contributions, debates, and future directions. Psychol. Sport Exerc. 80:102879. doi: 10.1016/j.psychsport.2025.102879, 40383282

[ref43] Oren-Yagoda R. Melamud-Ganani I. Aderka I. M. (2022). All by myself: loneliness in social anxiety disorder. J. Psychopathol. Clin. Sci. 131, 4–13. doi: 10.1037/abn0000705, 34843268

[ref44] Owen K. B. Manera K. E. Clare P. J. Lim M. H. Smith B. J. Phongsavan P. . (2024). Sport participation trajectories and loneliness: evidence from the longitudinal study of Australian children. J. Phys. Act. Health 21, 1341–1350. doi: 10.1123/jpah.2024-0319, 39304176

[ref45] Pels F. Kleinert J. (2016). Loneliness and physical activity: a systematic review. Int. Rev. Sport Exerc. Psychol. 9, 231–260. doi: 10.1080/1750984X.2016.1177849 PMC4706019 26807143

[ref46] Pickering L. Hadwin J. A. Kovshoff H. (2020). The role of peers in the development of social anxiety in adolescent girls: a systematic review. Adolesc. Res. Rev. 5, 341–362. doi: 10.1007/s40894-019-00117-x

[ref47] Plessner H. Ermark F. Schütz L.-M. Schweizer G. (2023). Sports performance judgments—an update from a social cognitive perspective. Asian J. Sport Exerc. Psychol. 3, 13–23. doi: 10.1016/j.ajsep.2023.01.002

[ref48] Qualter P. Rotenberg K. Barrett L. Henzi P. Barlow A. Stylianou M. . (2013). Investigating hypervigilance for social threat of lonely children. J. Abnorm. Child Psychol. 41, 325–338. doi: 10.1007/s10802-012-9676-x, 22956297

[ref49] Rangul V. Sund E. R. Ingul J. M. Rimehaug T. Pape K. Kvaløy K. (2024). Exploring the link between physical activity, sports participation, and loneliness in adolescents before and into the COVID-19 pandemic: the HUNT study, Norway. Int. J. Environ. Res. Public Health 21:1417. doi: 10.3390/ijerph21111417, 39595684 PMC11594226

[ref50] Ratnani I. J. (2017). Association of social anxiety disorder with depression and quality of life among medical undergraduate students. J. Family Med. Prim. Care 6, 243–248. doi: 10.4103/2249-4863.219992, 29302525 PMC5749064

[ref51] Reinwarth A. C. Beutel M. E. Schmidt P. Wild P. S. Münzel T. König J. . (2024). Loneliness and social anxiety in the general population over time—results of a cross-lagged panel analysis. Psychol. Med. 54, 4551–4560. doi: 10.1017/S0033291724001818, 39726175

[ref52] Ryan R. M. Deci E. L. (2020). Intrinsic and extrinsic motivation from a self-determination theory perspective: definitions, theory, practices, and future directions. Contemp. Educ. Psychol. 61:101860. doi: 10.1016/j.cedpsych.2020.101860

[ref53] Ryan R. M. Deci E. L. (2022). “Self-determination theory,” in Encyclopedia of Quality of Life and Well-Being Research, ed. Maggino F. (San Diego, CA: Springer International Publishing), 1–7.

[ref54] Salari N. Najafi H. Rasoulpoor S. Canbary Z. Heidarian P. Mohammadi M. (2025). The global prevalence and associated factors of loneliness in older adults: a systematic review and meta-analysis. Humanit. Soc. Sci. Commun. 12:985. doi: 10.1057/s41599-025-05304-x

[ref55] Shang Y. Chen S.-P. Liu L.-P. (2023). The role of peer relationships and flow experience in the relationship between physical exercise and social anxiety in middle school students. BMC Psychol. 11:428. doi: 10.1186/s40359-023-01473-z, 38057828 PMC10702045

[ref56] Shek D. T. L. (1994). Assessment of private and public self-consciousness: a Chinese replication. J. Clin. Psychol. 50, 341–348. doi: 10.1002/1097-4679(199405)50:3<341::AID-JCLP2270500305>3.0.CO;2-T, 8071439

[ref57] Shvedko A. Whittaker A. C. Thompson J. L. Greig C. A. (2018). Physical activity interventions for treatment of social isolation, loneliness or low social support in older adults: a systematic review and meta-analysis of randomised controlled trials. Psychol. Sport Exerc. 34, 128–137. doi: 10.1016/j.psychsport.2017.10.003

[ref58] Stein M. B. Kean Y. M. (2000). Disability and quality of life in social phobia: epidemiologic findings. Am. J. Psychiatry 157, 1606–1613. doi: 10.1176/appi.ajp.157.10.1606, 11007714

[ref59] Su D. L. Y. Wan A. W. L. Zhang L. Teng J. Chan D. K. C. (2024). Predicting adolescents’ leisure-time physical activity levels: a three-wave prospective test of the integrated model of self-determination theory and the theory of planned behavior. Behav. Sci. 14:693. doi: 10.3390/bs14080693, 39199089 PMC11351496

[ref60] Surkalim D. L. Clare P. J. Eres R. Gebel K. Bauman A. E. Ding D. (2024). Exercise to socialize? Bidirectional relationships between physical activity and loneliness in middle-aged and older American adults. Am. J. Epidemiol. 193, 996–1001. doi: 10.1093/aje/kwae001, 38319704 PMC11228862

[ref61] Surkalim D. L. Luo M. Eres R. Gebel K. van Buskirk J. Bauman A. . (2022). The prevalence of loneliness across 113 countries: systematic review and meta-analysis. BMJ 376:e067068. doi: 10.1136/bmj-2021-067068, 35140066 PMC8826180

[ref62] Teixeira P. J. Carraça E. V. Markland D. Silva M. N. Ryan R. M. (2012). Exercise, physical activity, and self-determination theory: a systematic review. Int. J. Behav. Nutr. Phys. Activity 9:78. doi: 10.1186/1479-5868-9-78, 22726453 PMC3441783

[ref63] Tella M. D. Adenzato M. Castelli L. Ghiggia A. (2023). Loneliness: association with individual differences in socioemotional skills. Personal. Individ. Differ. 203:111991. doi: 10.1016/j.paid.2022.111991

[ref64] Tubić T. Modrić T. Sekulić D. Bianco A. Radjo I. Drid P. (2023). Loneliness in sports active and non-active school-age children: can sport protect children against loneliness? Front. Psych. 13:1063714. doi: 10.3389/fpsyt.2022.1063714, 36713906 PMC9874288

[ref65] Üstündağ A. Haydaroğlu S. Sayan D. Güngör M. (2025). The relationship between social anxiety levels and effective communication skills of adolescents participating in sports. Sci. Rep. 15:15724. doi: 10.1038/s41598-025-00800-1, 40325045 PMC12053575

[ref66] Wan H. Huang W. Zhang W. Hu C. (2025). Exploring adolescents’ social anxiety, physical activity, and core self-evaluation: a latent profile and mediation approach. Int. J. Ment. Health Promot. 27, 1611–1626. doi: 10.32604/ijmhp.2025.070457

[ref67] White R. L. Bennie A. Vasconcellos D. Cinelli R. Hilland T. Owen K. B. . (2021). Self-determination theory in physical education: a systematic review of qualitative studies. Teach. Teach. Educ. 99:103247. doi: 10.1016/j.tate.2020.103247

[ref68] Wu D. Liu M. Li D. Yin H. (2024). The longitudinal relationship between loneliness and both social anxiety and mobile phone addiction among rural left-behind children: a cross-lagged panel analysis. J. Adolesc. 96, 969–982. doi: 10.1002/jad.12309, 38375869

[ref69] Wu J. Shao Y. Hu J. Zhao X. (2025a). The impact of physical exercise on adolescent social anxiety: the serial mediating effects of sports self-efficacy and expressive suppression. BMC Sports Sci. Med. Rehabil. 17:57. doi: 10.1186/s13102-025-01107-4, 40121514 PMC11929206

[ref70] Wu J. Shao Y. Zang W. Hu J. (2025b). Is physical exercise associated with reduced adolescent social anxiety mediated by psychological resilience?: evidence from a longitudinal multi-wave study in China. Child Adolesc. Psychiatry Ment. Health 19:17. doi: 10.1186/s13034-025-00867-8, 40045423 PMC11884043

[ref71] Xiao R. Du J. (2023). Reliability and validity of the 6-item UCLA loneliness scale (ULS-6) for application in adults. J. South. Med. Univ. 43, 900–905. doi: 10.12122/j.issn.1673-4254.2023.06.04, 37439161 PMC10339310

[ref72] Yang G. Li Y. Liu S. Liu C. Jia C. Wang S. (2021). Physical activity influences the mobile phone addiction among Chinese undergraduates: the moderating effect of exercise type. J. Behav. Addict. 10, 799–810. doi: 10.1556/2006.2021.00059, 34546969 PMC8997213

[ref73] Zhang S. Miao C. (2024). The mediating role of competence, autonomy, and relatedness in the activation and maintenance of sports participation behavior. Sci. Rep. 14:27124. doi: 10.1038/s41598-024-78760-1, 39511311 PMC11543691

[ref74] Zhao G. Sun K. Xue Y. Dong D. (2024). A chain-mediated model of the effect of physical exercise on loneliness. Sci. Rep. 14:30798. doi: 10.1038/s41598-024-81059-w, 39730531 PMC11681067

[ref75] Zika M. A. Becker L. (2021). Physical activity as a treatment for social anxiety in clinical and non-clinical populations: a systematic review and three Meta-analyses for different study designs. Front. Hum. Neurosci. 15:653108. doi: 10.3389/fnhum.2021.653108, 34177489 PMC8230570

