# Supplementary material for: Within-person reciprocal associations among physical activity, loneliness, and social anxiety in adolescence
Source: Front Psychol. 2026 Jul 2;17:1805256. doi: 10.3389/fpsyg.2026.1805256 (PMC13372597; doi:10.3389/fpsyg.2026.1805256)
Supplement: Supplementary file 1 [file Table_1.DOCX]

Supplementary Table 1 Skewness and kurtosis of main variables across four waves

| Variable | Skewness | Kurtosis |
| --- | --- | --- |
| PA at T1 | 0.58 | -1.09 |
| PA at T2 | 0.53 | -1.24 |
| PA at T3 | 0.64 | -1.21 |
| PA at T4 | 0.56 | -1.23 |
| SA at T1 | -0.06 | -0.56 |
| SA at T2 | -0.13 | -0.82 |
| SA at T3 | -0.18 | -0.89 |
| SA at T4 | -0.19 | -0.92 |
| Loneliness at T1 | 1.47 | 1.43 |
| Loneliness at T2 | 1.49 | 1.48 |
| Loneliness at T3 | 1.28 | 0.75 |
| Loneliness at T4 | 1.24 | 1.27 |
